# Efficacy of Airbo·Wave in Nighttime Compression for Leg Lymphedema

**DOI:** 10.3400/avd.oa.23-00084

**Published:** 2024-02-10

**Authors:** Kotaro Suehiro, Yukie Mizumoto, Noriyasu Morikage, Takasuke Harada, Yuriko Takeuchi, Soichi Ike, Ryunosuke Sakamoto, Ryo Suzuki, Hiroshi Kurazumi, Kimikazu Hamano

**Affiliations:** 1 Department of Surgery and Clinical Science, Yamaguchi University Graduate School of Medicine, Ube, Yamaguchi, Japan; 2 Department of Nursing, Yamaguchi University Hospital, Ube, Yamaguchi, Japan

**Keywords:** lymphedema, compression therapy, skin hardness

## Abstract

**Objectives:** This study aimed to clarify the efficacy of Airbo·Wave EV1 in nighttime compression therapy as part of complex decongestive therapy (CDT) for leg lymphedema.

**Patients and Methods:** We retrospectively reviewed 33 patients with leg lymphedema who used Airbo·Wave EV1 between April 2021 and September 2022. In these patients, the changes in leg volume and skin hardness were assessed using a scale ranging from 1 (softest) to 7 (hardest), and dermal thickness before and after the use of Airbo·Wave EV1 was evaluated.

**Results:** Twenty-two patients used Airbo·Wave EV1 for nighttime compression in CDT. Their skin hardness in the lower calf decreased mildly (mean scale: before, 3.9; after, 3.6 [*p* <0.05]), but the leg volume and skin thickness were unchanged. Eleven patients who were nonadherent could restart compression therapy by using Airbo·Wave EV1. Their skin hardness in the medial lower calf (before, 5.1; after, 4.3 [*p* <0.05]), leg volume (before, 8412 mL; after, 8191 mL [*p* <0.01]), and skin thickness in the medial and lateral lower leg were reduced.

**Conclusion:** Airbo·Wave EV1 could improve skin hardness in the calf area. Moreover, it is a safe procedure for the nonadherent while reducing leg volume reasonably.

## Introduction

**Figure 1:**
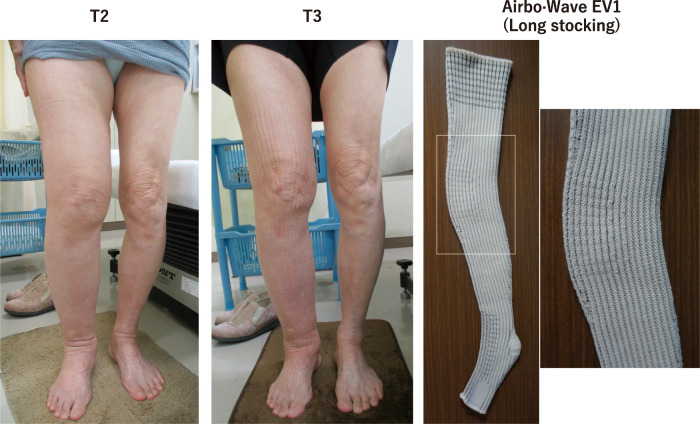
Fig. 1 Images of the right leg with lymphedema and Airbo·Wave EV1. The change in the right leg before (left image, T2) and after (middle image, T3) the use of Airbo·Wave EV1 is demonstrated. The longitudinally running striped mark on the skin is caused by the texture of Airbo·Wave EV1 (right image) after removal.

The primary treatment for peripheral lymphedema is complex decongestive therapy (CDT), which involves a two-stage treatment program. Compression therapy is typically provided using multilayered bandages in the first phase, whereas medical compression stocking is used in the second phase.^1)^ The adoption of nighttime compression in the second phase is a growing trend because it allows for the better management of edema, pain, a sense of relief, etc.^2)^ Compared with daytime compression, nighttime compression is less restrictive, easy to apply, and encourages airflow while maintaining a reasonable degree of compression.^2)^ Airbo·Wave EV1 (Sanyu Medical Co., Ltd., Aichi, Japan) is a compression stocking for nighttime compression (**Fig. 1**). According to the brochure, this compression garment has unique features. First, it is made of flat-knitted cotton fabric and is combined with a polyester mesh to improve aeration, comfort, and ease of application. Second, it has waves running longitudinally that aims to promote lymph flow. Finally, it provides 20–25 mmHg of interface pressure uniformly but is not designed to create graduated compression. Given that we recently encountered a series of patients with leg lymphedema who preferentially used Airbo·Wave EV1 for nighttime compression, we reviewed their changes in leg volumes and skin hardness to study the efficacy of Airbo·Wave EV1 in CDT.

## Patients and Methods

This retrospective study was approved by the Institutional Review Board of Yamaguchi University Hospital (Ube, Yamaguchi, Japan; approval no. 2022-108), and the need for individual informed consent was waived. A total of 265 patients with leg lymphedema first visited our clinic between April 2009 and March 2023; the patients were also followed up in our clinic. For all patients, CDT was provided as recommended by the International Society of Lymphology.^1)^ A total of 33 patients with unilateral leg lymphedema started using Airbo·Wave EV1 after April 2021. **Table 1** shows the patient characteristics. Twenty-two patients used Airbo·Wave EV1 for nighttime compression as part of CDT (group 1). The other 11 patients discontinued compression therapy for a certain period mainly because of old age and weakness, which prevented them from applying and tolerating compression garments. However, they restarted compression therapy with Airbo·Wave EV1 because of its ease of application and comfort (group 2). All patients in group 2 used Airbo·Wave EV1 not only for nighttime compression but also for daytime compression spontaneously. The changes in their leg conditions were assessed in the below time points:

T1: 6–12 months prior to the use of Airbo Wave EV1.
T2: Less than 1 month prior to the use of Airbo Wave EV1.
T3: Following 3–6 months of regular use of Airbo Wave EV1.

**Table:** Table 1 Patient characteristics

Characteristics	Group 1 (n = 22)	Group 2 (n = 11)
Age (years)	62 (40–87)	76 (63–89)
Female–male ratio	21:1	10:1
Body mass index (kg/m^2^)	26 (18–34)	21 (18–29)
Affected leg (right–left ratio)	7:15	7:4
Primary–secondary ratio	1:21	1:10
ISL stage (I–II–III ratio)	0:20:2	0:10:1
Compression stockings		(Before abandoning compression therapy)
**Daytime stockings**		
mediven forte CCL3^a^	5	2
mediven plus CCL3^a^	4	2
Essential Thermoregulating CCL3^b^	3	0
mediven forte CCL2^a^	4	3
Essential Thermoregulating CCL2^b^	2	1
mediven plus CCL2^a^	1	0
Prima M35 (CCL3)^c^	2	1
JOBST Elvarex CCL3^d^	1	0
JOBST OPAQUE CCL2^d^	0	1
Multilayer bandages	0	1
**Nighttime compression before the use of Airbo·Wave EV1**		
None	10	9
Multilayer bandages	7	2
Mobiderm Autofit^e^	4	0
JOBST Relax^d^	1	0

Group 1: patients who used Airbo·Wave EV1 as an adjunctive for night care; group 2: patients who used Airbo·Wave EV1 as a main compression stocking.

a: medi GmbH & Co. KG, Bayreuth, Germany

b: SIGVARIS AG, St. Gallen, Switzerland

c: Medix Co., Ltd., Tokushima, Japan

d: BSN-JOBST GmbH, Emmerich am Rhein, Germany

e: THUASNE SAS, Levallois-Perret, France

ISL: the International Society of Lymphology

The leg circumferences were measured in 10 cm intervals from the ankle to the groin, and the leg volume was calculated using the truncated cone method.

For the assessment of skin hardness, we used a palpation scale (P-scale) as previously reported.^3)^ The scale is composed of three commercial sponges (Yahata Neji Corporation, Aichi, Japan) attached to a board, and the score ranges from 1 (softest) to 7 (hardest). The softest sponge (P-scale = 2) was as soft as the skin of a normal inner thigh, and the hardest sponge (P-scale = 6) was as hard as the skin with lipodermatosclerosis. The medium sponges (P-scale = 4) had a hardness level that was in between the softest and hardest sponges. On the P-scale, a score of 1 was softer than 2, 3 was between 2 and 4, 5 was between 4 and 6, and 7 was harder than 6. The P-scale was always determined on the basis of the agreement between patients and their therapist.

Skin ultrasonography was performed using the Xario 200G ultrasound system (Canon Medical Systems Corporation, Tochigi, Japan) with a 7 to 11 MHz linear transducer. Eight points in a leg (middle of the medial/lateral and upper/lower of the thigh and calf) were scanned. The thickness of the structures was defined as previously reported^4)^:

- Dermis: the distance between the posterior echogenic border of the epidermal entrance echo and the posterior echogenic border of the dermis.
- Subepidermal low-echogenic band (SLEB): the width of the echo-free space in the dermis.

Considering that the borders of these structures were irregular and not always located parallel to the epidermal surface, the distances were measured at three points of each acquired image and then averaged.

### Statistical analysis

**Figure 2:**
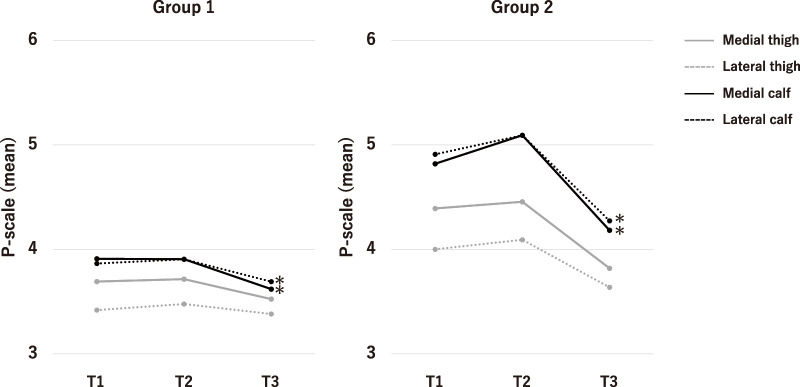
Fig. 2 The changes in skin hardness assessed using a P-scale. *p <0.05, compared with T2.

The results are expressed as median (range) or count unless otherwise indicated. Although the number of patients is small, the changes in P-scales (**Fig. 2**) are shown using mean values because the changes are blurred when they are expressed using median values. The Wilcoxon signed rank-sum test was used to evaluate the differences in measurements in the same leg. The Mann–Whitney *U* test was used to evaluate the between-group differences. The χ^2^ test was used to test the categorical variables, including the changes in P-scales. Statistical analyses were performed using JMP 11.0 (SAS Institute, Cary, NC, USA), and *p* <0.05 was considered statistically significant.

## Results

### Effect of Airbo·Wave EV1 in group 1

Patients in group 1 had been undergoing CDT for 6.1 years (1.2–11.6 years) before the use of Airbo·Wave EV1. **Table 1** lists the types of compression stockings used for daytime care. Ten patients (45%) had not used nighttime compression, and seven patients (32%) had used multilayer bandages for nighttime care before using Airbo·Wave EV1. Twenty-two patients (95%) used the long-stocking Airbo·Wave EV1, whereas only one patient used the knee-high Airbo·Wave EV1. Following 4.9 months (3.0–5.5 months) of regular use of Airbo·Wave EV1, no significant changes in leg volume, circumferences, and dermal thickness were observed (**Table 2**). The P-scales in the calf area of 9 patients (41%) decreased by 1–2 but were unchanged in the remaining 13 patients. As a result, mild but significant decreases in mean P-scales were confirmed in the medial and lateral calf (**Fig. 2**). No exacerbation was observed in terms of skin hardness. These results were similarly observed in the patients who had been using night care and those who had not.

**Table:** Table 2 Measurements

	Group 1 (N = 22)			Group 2 (N = 11)		
	T1	T2	T3	T1	T2	T3
Leg volume (mL)	7569 (5569–10377)	7488 (5481–11362)	7565 (4949–12472)	8188 (4080–10825)^*^	8412 (6276–11623)	8191 (5271–11358)^*^
Circumference (cm)						
20 cm above the patella	54.7 (47.5–66.1)	55.4 (47.3–72.5)	55.9 (45.8–76.0)	53.2 (44.2–62.7)	53.2 (48.0–58.2)	51.0 (46.7–58.0)^*^
Upper edge of the patella	41.1 (37.0–48.2)	42.9 (36.5–56.3)	42.5 (34.5–55.5)	43.7 (34.5–55.0)	44.8 (39.5–55.2)	43.3 (38.1–51.2)^*^
30 cm below the patella	24.3 (21.1–32.5)	24.8 (21.1–35.8)	25.0 (20.5–35.6)	28.2 (22.2–40.3)	28.8 (22.5–40.3)	28.2 (21.0–37.6)^*^
Foot	22.3 (21.1–25.5)	22.2 (20.3–25.3)	21.7 (20.0–25.5)	22.8 (21.0–25.6)	22.8 (21.2–25.7)	23.1 (20.9–25.2)
Dermis (mm)						
Medial upper thigh	2.1 (1.4–4.4)	2.2 (1.1–4.4)	2.3 (1.1–4.1)	2.2 (1.4–3.8)	2.2 (1.6–3.8)	2.7 (1.1–4.1)
Lateral upper thigh	2.2 (1.0–4.1)	2.2 (1.0–4.1)	2.3 (1.1–3.8)	2.2 (2.0–3.8)	2.8 (2.5–4.1)	3.3 (2.0–3.9)
Medial lower thigh	2.7 (1.8–4.4)	2.8 (1.8–3.1)	2.6 (1.4–3.8)	2.7 (1.8–3.9)	3.1 (2.3–4.4)	3.6 (1.6–4.1)
Lateral lower thigh	2.2 (0.9–3.4)	2.1 (1.1–3.4)	2.2 (1.1–3.4)	2.3 (1.1–3.5)	2.3 (2.2–3.9)	2.8 (1.7–4.1)
Medial upper calf	2.3 (0.5–3.6)	2.3 (0.5–3.8)	2.5 (0.8–3.6)	3.0 (1.6–3.8)	3.6 (1.9–4.1)	3.1 (2.3–4.7)
Lateral upper calf	2.0 (0.5–5.4)	2.0 (0.7–5.3)	2.1 (0.8–3.8)	2.7 (2.2–4.1)	3.1 (2.3–4.7)	2.7 (1.9–4.7)
Medial lower calf	2.6 (0.8–4.3)	2.7 (0.8–4.4)	2.7 (1.0–4.4)	3.0 (2.2–4.1)^*^	3.8 (2.7–4.2)	3.1 (2.5–4.1)^*^
Lateral lower calf	2.2 (1.0–3.9)	2.1 (0.7–3.8)	2.0 (1.1–3.8)	3.0 (1.6–5.2)^*^	3.8 (2.2–4.4)	3.1 (1.9–5.0)^*^
SLEB (mm)						
Dorsum of the foot	1.8 (0.5–2.5)	1.8 (0.7–2.7)	1.7 (0.6–2.8)	2.3 (1.6–4.1)	2.0 (1.6–4.1)	2.2 (1.2–3.6)
Medial upper thigh	0.7 (0.3–1.4)	0.8 (0.0–0.9)	0.9 (0.0–1.6)	0.9 (0.5–1.5)	1.1 (0.6–1.4)	1.0 (0.5–1.4)
Lateral upper thigh	0.3 (0.0–1.2)	0.2 (0.0–4.4)	0.2 (0.0–1.0)	0.9 (0.0–2.0)	0.9 (0.1–1.1)	0.9 (0.7–1.3)
Medial lower thigh	0.6 (0.3–1.6)	0.7 (0.1–1.7)	0.7 (0.0–2.0)	1.1 (1.0–2.0)	1.1 (1.0–2.0)	0.9 (0.1–1.9)
Lateral lower thigh	0.4 (0.1–1.1)	0.3 (0.1–1.0)	0.2 (0.0–0.9)	1.0 (0.4–1.3)	1.0 (0.2–1.4)	0.7 (0.1–1.3)
Medial upper calf	0.3 (0.0–1.1)	0.4 (0.1–1.4)	0.5 (0.0–1.1)	1.3 (0.4–1.9)	1.3 (0.8–2.0)	1.3 (0.6–1.6)
Lateral upper calf	0.5 (0.0–0.9)	0.4 (0.1–1.7)	0.2 (0.0–1.1)	0.9 (0.4–2.2)	0.9 (0.2–2.4)	1.1 (0.1–1.3)
Medial lower calf	0.5 (0.3–1.3)	0.5 (0.1–1.6)	0.6 (0.0–1.7)	1.1 (0.4–2.9)^*^	1.8 (1.3–1.9)	1.2 (0.6–1.3)^*^
Lateral lower calf	0.5 (0.3–0.9)	0.4 (0.1–1.9)	0.4 (0.1–1.1)	0.8 (0.2–3.0)^*^	1.3 (0.7–1.7)	0.9 (0.3–1.4)^*^
Dorsum of the foot	0.4 (0.0–1.0)	0.3 (0.1–1.3)	0.5 (0.1–1.0)	0.5 (0.2–1.9)	1.1 (0.1–1.3)	0.8 (0.5–1.5)

^*^p <0.05 vs. pre.

SLEB: subepidermal low-echogenic band

### Effect of Airbo·Wave EV1 in group 2

**Figure 3:**
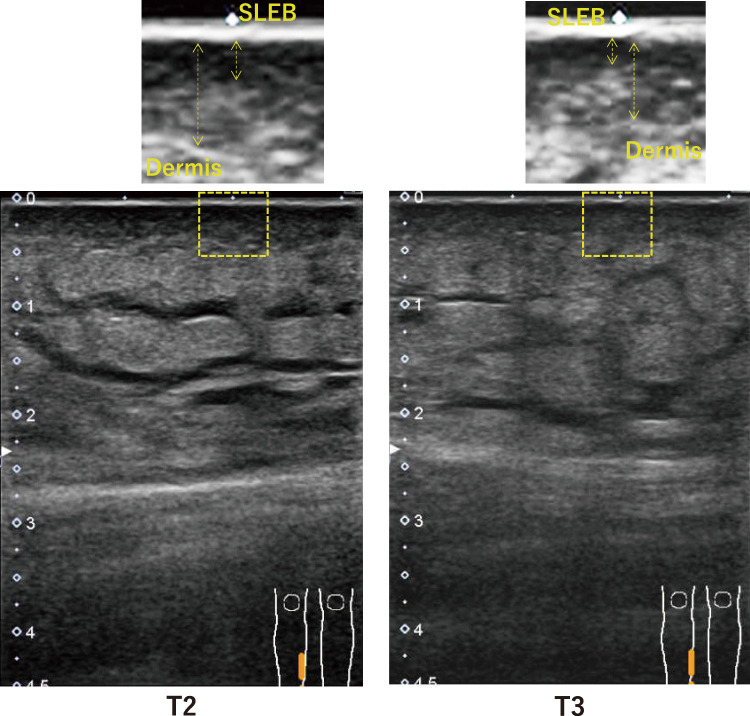
Fig. 3 Representative ultrasonography images of the medial lower calf of a patient in group 2 before (T2) and after (T3) the use of Airbo·Wave EV1. Both dermal and SLEB thickness are reduced after the use of Airbo·Wave EV1. Note that a significant amount of subcutaneous echo-free space, i.e., subcutaneous edema, remains even after the use of Airbo·Wave EV1. SLEB: subepidermal low-echogenic band

Patients in group 2 were significantly older than those in group 1 (*p* <0.01) and had been undergoing CDT for 7.9 years (1.0–10.8 years) before discontinuing compression therapy. Five patients (45%) could not tolerate or had difficulty applying class 2 compression stockings (**Table 1**). They had been free from compression therapy for 1.3 years (0.6–3.0 years) before using Airbo·Wave EV1. Eight patients (73%) used long-stocking Airbo·Wave EV1, whereas three patients used knee-high Airbo·Wave EV1. Eleven patients (91%) adhered well to the use of Airbo·Wave EV1 and preferred to use it day and night. One patient could not tolerate the compression level and refused compression therapy completely. Following 4.4 months (3.0–6.0 months) of regular use of Airbo·Wave EV1, significant reductions in leg volume and circumferences in the thigh and calf were observed (**Table 2**); however, a certain amount of edema still remained (**Fig. 3**). The P-scales in the calf area of seven patients (64%) decreased by one to two but were unchanged in the remaining four patients. As a result, significant decreases in mean P-scales were observed in the medial and lateral calf (**Fig. 2**). Decreases were also observed in dermal and SLEB thickness in the corresponding area (**Table 2**). No exacerbation was observed in terms of skin hardness.

## Discussion

The main findings of this study are as follows: (1) Skin hardness in the calf area improved by using Airbo·Wave EV1. (2) No additional reduction in leg volume was obtained in patients who were already undergoing CDT. (3) Patients who could not tolerate compression therapy adhered well to the use of Airbo·Wave EV1, and a reasonable reduction in their leg volume was achieved.

### Skin softening effect by Airbo·Wave EV1

Whitaker reported that 45% of patients with peripheral lymphedema who were already on CDT experienced a further reduction of edema by adding nighttime compression therapy.^5)^ However, this was not confirmed in the current study. Instead, a skin-softening effect was confirmed particularly in the calf area after using Airbo·Wave EV1. We previously reported that the skin hardness sensed via skin palpation in legs with lymphedema was predominantly affected by pathological dermal thickening.^3)^ This thickening is due to the increased accumulation of water in the dermis, which can be confirmed as increased SLEB thickness, and collagen content.^6)^ In the current study, the reduction of skin hardness in the calf area of group 2 was associated with reductions in dermal and SLEB thicknesses. Therefore, it was assumed that the decrease in water in the dermis after using Airbo·Wave EV1 partly contributed to the improved skin hardness in these areas. However, in group 1, a mild improvement of skin hardness was achieved in the calf area, whereas the dermal and SLEB thicknesses were unchanged. A possible explanation would be that the uneven surface of Airbo·Wave EV1 exerted a mechanical effect on the skin, but this assumption needs to be confirmed in future studies.

### Compression therapy for the nonadherent

Difficulty in donning/doffing and discomfort are the primary reasons for nonadherence to compression therapy.^7)^ Compression garments for nighttime care are designed to be easy to apply and minimally restrictive. These are particularly beneficial for weak elderly people who have problems in applying and tolerating compression garments. Indeed, 91% of patients in group 2 who had been nonadherent became able to undergo 24-hour compression by using Airbo·Wave EV1. In these patients, a significant but insufficient reduction in leg volume was confirmed. The significance of such edema reduction should be studied in terms of prognosis, quality of life, etc., in future studies. However, this type of compromise is inevitable in the management of lymphedema in weak elderly patients.

### Limitations

Considering that this study was a single-center retrospective study that included a small number of subjects, reaching a definite conclusion was challenging. We could only use low-resolution ultrasonography, which was insufficient for the evaluation of thin structures such as the dermis and SLEB. In addition, the P-scale is a subjective measure. This is inevitable because there are currently no appropriate methods for measuring skin hardness that represent well the experiences of physicians, therapists, and patients. These might affect the evaluation of the relation between skin structure and hardness.

## Conclusion

We found that skin hardening in the calf could be improved by using Airbo·Wave EV1. In addition, continued compression therapy and subsequent significant leg volume reduction could be achieved among patients who had been nonadherent to compression therapy by using Airbo·Wave EV1.

## Acknowledgments

This research received no specific grant from any funding agency in the public, commercial, or not-for-profit sectors.

## Disclosure Statement

The authors have no conflict of interests to declare.

## Author Contributions

Study conception: KS

Data collection: KS, YM, TH, YT, SI, RS, RS, and HK

Analysis: KS

Investigation: KS

Writing: KS

Funding acquisition: NM and KH

Critical review and revision: all authors

Final approval of the article: all authors

Accountability for all aspects of the work: all authors.
